# Supplementary material for: Machine Learning Accelerates Crystallization for Structure Determination
Source: Angew Chem Int Ed Engl. 2026 May 4;65(25):e1218503. doi: 10.1002/anie.1218503 (PMC13266958; doi:10.1002/anie.1218503)

checkCIF/PLATON report

Structure factors have been supplied for datablock(s) lcz051

THIS REPORT IS FOR GUIDANCE ONLY. IF USED AS PART OF A REVIEW PROCEDURE FOR PUBLICATION, IT SHOULD NOT REPLACE THE EXPERTISE OF AN EXPERIENCED CRYSTALLOGRAPHIC REFEREE.

No syntax errors found. CIF dictionary Interpreting this report

Datablock: lcz051

Bond precision:	C-C = 0.0242 A		Wavelength=1.54184
Cell:	a=14.9707(3)	b=15.6255(4)	c=23.4814(5)
	alpha=101.863(2)	beta=104.219(2)	gamma=106.042(2)
Temperature:	100 K		

	Calculated	Reported
Volume	4891.9(2)	4891.8(2)
Space group	P -1	P -1
Hall group	-P 1	-P 1
Moiety formula	C15 H3 Ag3 F18 N6, C11 H8 O	C15 H3 Ag3 F18 N6, C11 H8 O
Sum formula	C26 H11 Ag3 F18 N6 O	C26 H11 Ag3 F18 N6 O
Mr	1089.02	1089.02
Dx,g cm-3	2.218	2.218
Z	6	6
Mu (mm-1)	15.608	15.608
F000	3120.0	3120.0
F000'	3135.18	
h,k,lmax	17,18,27	17,18,27
Nref	17291	17143
Tmin,Tmax	0.043,0.082	0.282,1.000
Tmin'	0.007	

Correction method= # Reported T Limits: Tmin=0.282 Tmax=1.000
AbsCorr = MULTI-SCAN

Data completeness= 0.991 Theta(max)= 66.599

R(reflections)= 0.0984(12830) wR2(reflections)= 0.2338(17143)

S = 1.033 Npar= 1405

The following ALERTS were generated. Each ALERT has the format
test-name_ALERT_alert-type_alert-level.
Click on the hyperlinks for more details of the test.

Alert level A

PLAT971_ALERT_2_A Check Calcd Resid. Dens. 1.05Ang From Ag09 4.16 eA-3

Author Response: This Alert is due to presence of residual density in the presence of heavy metal atom (Ag)

Alert level B

PLAT342_ALERT_3_B Low Bond Precision on C-C Bonds .... 0.02422 Ang.

Author Response: Disordered structure.

Alert level C

```
DIFMX02_ALERT_1_C  The maximum difference density is > 0.1*ZMAX*0.75
            The relevant atom site should be identified.
PLAT094_ALERT_2_C Ratio of Maximum / Minimum Residual Density ....       2.11 Report
PLAT097_ALERT_2_C Large Reported Max.  (Positive) Residual Density       4.42 eA-3
PLAT220_ALERT_2_C NonSolvent   Resd 3  C   Ueq(max)/Ueq(min) Range        3.9 Ratio
PLAT234_ALERT_4_C Large Hirshfeld Difference F00Q     --C03S     .       0.18 Ang.
PLAT234_ALERT_4_C Large Hirshfeld Difference F00V     --C02W     .       0.18 Ang.
PLAT234_ALERT_4_C Large Hirshfeld Difference F01H     --C049     .       0.16 Ang.
PLAT234_ALERT_4_C Large Hirshfeld Difference F01F     --C03R     .       0.16 Ang.
PLAT234_ALERT_4_C Large Hirshfeld Difference F03E     --C032     .       0.24 Ang.
PLAT234_ALERT_4_C Large Hirshfeld Difference F02M     --C03N     .       0.19 Ang.
PLAT234_ALERT_4_C Large Hirshfeld Difference F02P     --C046     .       0.25 Ang.
PLAT234_ALERT_4_C Large Hirshfeld Difference F02X     --C03N     .       0.23 Ang.
PLAT234_ALERT_4_C Large Hirshfeld Difference C036     --C044     .       0.21 Ang.
PLAT241_ALERT_2_C High   'MainMol' Ueq as Compared to Neighbors of       C03B Check
PLAT241_ALERT_2_C High   'MainMol' Ueq as Compared to Neighbors of       C043 Check
PLAT241_ALERT_2_C High   'MainMol' Ueq as Compared to Neighbors of       C04N Check
PLAT241_ALERT_2_C High   'MainMol' Ueq as Compared to Neighbors of       C04B Check
PLAT242_ALERT_2_C Low    'MainMol' Ueq as Compared to Neighbors of       C03X Check
PLAT309_ALERT_2_C Single Bonded Oxygen (C-O > 1.3 Ang) ..........       O024 Check
PLAT369_ALERT_2_C Long   C(sp2)-C(sp2) Bond  C2        - C03H     .       1.55 Ang.
PLAT906_ALERT_3_C Large K Value in the Analysis of Variance ......     25.502 Check
PLAT906_ALERT_3_C Large K Value in the Analysis of Variance ......      5.241 Check
PLAT906_ALERT_3_C Large K Value in the Analysis of Variance ......      2.563 Check
```

```
PLAT911_ALERT_3_C Missing FCF Refl Between Thmin & STh/L=    0.595        147 Report
               10  9  0,  11  9  0,   7 10  0,   8 10  0,  10 10  0,   9 11  0,
               -9-11  1,  -7-11  1,  -6-11  1, -10-10  1,  -8-10  1, -11 -9  1,
              -10 -9  1, -12 -8  1,  17 -7  1, -12  4  1,   6 11  1,   7 11  1,
                9 11  1,   6 12  1,   1 14  1,   2 16  1,  -7 17  1,  -6-14  2,
               -9-11  2,  -7-11  2, -10-10  2,  -8-10  2, -11 -9  2, -10 -9  2,
                   (  117 More Missing: see the .ckf listing file)
PLAT971_ALERT_2_C Check Calcd Resid. Dens.  0.77Ang From Ag08           2.42 eA-3
```

Author Response: This Alert is due to presence of residual density in the presence of heavy metal atom (Ag)

```
PLAT971_ALERT_2_C Check Calcd Resid. Dens.  1.28Ang From F041           2.24 eA-3
```

Author Response: This Alert is due to presence of residual density in the presence of heavy metal atom (Ag)

```
PLAT971_ALERT_2_C Check Calcd Resid. Dens.  1.23Ang From C03B           2.11 eA-3
```

Author Response: This Alert is due to presence of residual density in the presence of heavy metal atom (Ag)

```
PLAT971_ALERT_2_C Check Calcd Resid. Dens.  1.44Ang From C028           2.09 eA-3
```

Author Response: This Alert is due to presence of residual density in the presence of heavy metal atom (Ag)

```
PLAT971_ALERT_2_C Check Calcd Resid. Dens.  1.12Ang From F04I           2.05 eA-3
```

Author Response: This Alert is due to presence of residual density in the presence of heavy metal atom (Ag)

```
PLAT971_ALERT_2_C Check Calcd Resid. Dens.  0.98Ang From F041           1.94 eA-3
```

Author Response: This Alert is due to presence of residual density in the presence of heavy metal atom (Ag)

```
PLAT971_ALERT_2_C Check Calcd Resid. Dens.  1.19Ang From O01N           1.94 eA-3
```

Author Response: This Alert is due to presence of residual density in the presence of heavy metal atom (Ag)

```
PLAT971_ALERT_2_C Check Calcd Resid. Dens.  1.15Ang From F04F           1.81 eA-3
```

Author Response: This Alert is due to presence of residual density in the presence of heavy metal atom (Ag)

PLAT971_ALERT_2_C Check Calcd Resid. Dens. 0.30Ang From C02Z 1.81 eA-3

Author Response: This Alert is due to presence of residual density in the presence of heavy metal atom (Ag)

PLAT971_ALERT_2_C Check Calcd Resid. Dens. 0.74Ang From Ag07 1.79 eA-3

Author Response: This Alert is due to presence of residual density in the presence of heavy metal atom (Ag)

PLAT971_ALERT_2_C Check Calcd Resid. Dens. 1.20Ang From C03B 1.77 eA-3

Author Response: This Alert is due to presence of residual density in the presence of heavy metal atom (Ag)

PLAT971_ALERT_2_C Check Calcd Resid. Dens. 0.92Ang From C047 1.73 eA-3

Author Response: This Alert is due to presence of residual density in the presence of heavy metal atom (Ag)

PLAT971_ALERT_2_C Check Calcd Resid. Dens. 1.24Ang From F04C 1.69 eA-3

Author Response: This Alert is due to presence of residual density in the presence of heavy metal atom (Ag)

PLAT971_ALERT_2_C Check Calcd Resid. Dens. 0.83Ang From C048 1.67 eA-3

Author Response: This Alert is due to presence of residual density in the presence of heavy metal atom (Ag)

PLAT971_ALERT_2_C Check Calcd Resid. Dens. 1.15Ang From F03A 1.65 eA-3

Author Response: This Alert is due to presence of residual density in the presence of heavy metal atom (Ag)

PLAT971_ALERT_2_C Check Calcd Resid. Dens. 1.19Ang From F027 1.61 eA-3

Author Response: This Alert is due to presence of residual density in the presence of heavy metal atom (Ag)

```
PLAT971_ALERT_2_C Check Calcd Resid. Dens.  0.93Ang From C01I           1.59 eA-3
```

Author Response: This Alert is due to presence of residual density in the presence of heavy metal atom (Ag)

```
PLAT971_ALERT_2_C Check Calcd Resid. Dens.  0.91Ang From C047           1.59 eA-3
```

Author Response: This Alert is due to presence of residual density in the presence of heavy metal atom (Ag)

```
PLAT971_ALERT_2_C Check Calcd Resid. Dens.  0.79Ang From C3             1.57 eA-3
```

Author Response: This Alert is due to presence of residual density in the presence of heavy metal atom (Ag)

```
PLAT971_ALERT_2_C Check Calcd Resid. Dens.  0.69Ang From C028           1.52 eA-3
```

Author Response: This Alert is due to presence of residual density in the presence of heavy metal atom (Ag)

```
PLAT971_ALERT_2_C Check Calcd Resid. Dens.  1.11Ang From Ag03           1.51 eA-3
```

Author Response: This Alert is due to presence of residual density in the presence of heavy metal atom (Ag)

```
PLAT972_ALERT_2_C Check Calcd Resid. Dens.  0.85Ang From Ag08          -2.43 eA-3
PLAT972_ALERT_2_C Check Calcd Resid. Dens.  0.69Ang From Ag09          -1.81 eA-3
PLAT972_ALERT_2_C Check Calcd Resid. Dens.  0.82Ang From Ag07          -1.53 eA-3
PLAT977_ALERT_2_C Check Negative Difference Density on H028      .     -0.37 eA-3
PLAT977_ALERT_2_C Check Negative Difference Density on H048      .     -0.50 eA-3
PLAT977_ALERT_2_C Check Negative Difference Density on H043      .     -0.48 eA-3
PLAT977_ALERT_2_C Check Negative Difference Density on H04N      .     -0.63 eA-3
PLAT977_ALERT_2_C Check Negative Difference Density on H031      .     -0.34 eA-3
```

Alert level G

```
PLAT002_ALERT_2_G Number of Distance or Angle Restraints on AtSite          6 Note
PLAT003_ALERT_2_G Number of Uiso or U(i,j)  Restrained non-H-Atoms        162 Report
PLAT083_ALERT_2_G SHELXL Second Parameter in WGHT  Unusually Large     136.99 Why ?
PLAT154_ALERT_1_G The s.u.'s on the Cell Angles are Equal ..(Note)      0.002 Degree
PLAT171_ALERT_4_G The CIF-Embedded .res File Contains EADP Records          1 Report
PLAT172_ALERT_4_G The CIF-Embedded .res File Contains DFIX Records          5 Report
PLAT177_ALERT_4_G The CIF-Embedded .res File Contains DELU Records          8 Report
PLAT178_ALERT_4_G The CIF-Embedded .res File Contains SIMU Records         10 Report
PLAT186_ALERT_4_G The CIF-Embedded .res File Contains ISOR Records          3 Report
PLAT188_ALERT_3_G A Non-default SIMU Restraint Value has been used     0.0100 Report
PLAT188_ALERT_3_G A Non-default SIMU Restraint Value has been used     0.0100 Report
PLAT188_ALERT_3_G A Non-default SIMU Restraint Value has been used     0.0200 Report
PLAT188_ALERT_3_G A Non-default SIMU Restraint Value has been used     0.0100 Report
```

```
PLAT192_ALERT_3_G A Non-default DELU Restraint Value for SecondPar      0.0200 Report
PLAT192_ALERT_3_G A Non-default DELU Restraint Value for SecondPar      0.0200 Report
PLAT242_ALERT_2_G Low    'MainMol' Ueq as Compared to Neighbors of        C02W Check
PLAT242_ALERT_2_G Low    'MainMol' Ueq as Compared to Neighbors of        C03G Check
PLAT242_ALERT_2_G Low    'MainMol' Ueq as Compared to Neighbors of        C03K Check
PLAT242_ALERT_2_G Low    'MainMol' Ueq as Compared to Neighbors of        C03Q Check
PLAT242_ALERT_2_G Low    'MainMol' Ueq as Compared to Neighbors of        C03S Check
PLAT242_ALERT_2_G Low    'MainMol' Ueq as Compared to Neighbors of        C049 Check
PLAT242_ALERT_2_G Low    'MainMol' Ueq as Compared to Neighbors of        C02A Check
PLAT242_ALERT_2_G Low    'MainMol' Ueq as Compared to Neighbors of        C032 Check
PLAT242_ALERT_2_G Low    'MainMol' Ueq as Compared to Neighbors of        C037 Check
PLAT242_ALERT_2_G Low    'MainMol' Ueq as Compared to Neighbors of        C03I Check
PLAT242_ALERT_2_G Low    'MainMol' Ueq as Compared to Neighbors of        C03R Check
PLAT242_ALERT_2_G Low    'MainMol' Ueq as Compared to Neighbors of        C04H Check
PLAT242_ALERT_2_G Low    'MainMol' Ueq as Compared to Neighbors of        C03C Check
PLAT242_ALERT_2_G Low    'MainMol' Ueq as Compared to Neighbors of        C03N Check
PLAT242_ALERT_2_G Low    'MainMol' Ueq as Compared to Neighbors of        C046 Check
PLAT242_ALERT_2_G Low    'MainMol' Ueq as Compared to Neighbors of        C04A Check
PLAT242_ALERT_2_G Low    'MainMol' Ueq as Compared to Neighbors of        C04L Check
PLAT434_ALERT_2_G Short Inter HL..HL Contact F00E     ..F02X     .        2.84 Ang.
                                                1-x,2-y,1-z  =      2_676 Check
PLAT434_ALERT_2_G Short Inter HL..HL Contact F00M     ..F01Q     .        2.81 Ang.
                                                      x,y,z  =      1_555 Check
PLAT434_ALERT_2_G Short Inter HL..HL Contact F00S     ..F013     .        2.83 Ang.
                                                    x,1+y,z  =      1_565 Check
PLAT434_ALERT_2_G Short Inter HL..HL Contact F027     ..F03F     .        2.84 Ang.
                                                1-x,2-y,2-z  =      2_677 Check
PLAT720_ALERT_4_G Number of Unusual/Non-Standard Labels ..........         188 Note
              Ag01    Ag02    Ag03    Ag04    Ag05    Ag06    Ag07    Ag08
              Ag09    N00A    N00B    N00C    N00D    F00E    N00F    N00G
              N00H    N00I    N00J    N00K    F00L    F00M    F00N    N00O
              N00P    F00Q    N00R    F00S    F00T    F00U    F00V    F00W
              N00X    N00Y    F00Z    F010    F011    N012    F013    N014
              F015    N016    F017    O018    F019    F01A    F01B    F01C
              F01D    F01E    F01F    F01G    F01H    C01J    H01J    F01K
              C01L    H01L    C01M    H01M    O01N    F01O    F01P    F01Q
              F01R    C01S    C01T    H01T    F01U    C01V    C01W    F01X
              C01Y    H01Y    C01Z    F020    C021    C022    C023    O024
              F025    C026    F027    C028    H028    F029    C02A    C02B
              H02B    C02C    H02C    C02D    C02E    C02F    H02F    C02G
              C02H    H02H    C02I    C02K    H02K    C02L    F02M    C02N
              C02O    H02O    F02P    C02Q    C02R    F02T    F02U    F02V
              C02W    F02X    C02Y    H02Y    F030    C032    C033    H033
              C034    F035    C036    C037    F03A    C03B    H03B    C048
              H048    C043    H043    C01I    C03X    C03P    H03P    C03L
              H03L    C03C    C03D    F03E    F03F    C03G    C03H    H03H
              C03I    C03J    C03K    C03N    C03O    H03O    C03Q    C03R
              C03S    C03U    F03V    C03W    H03W    F041    F042    C044
              F045    C046    C049    C04A    C04B    H04B    F04C    F04F
              C04H    F04I    C04L    C04N    H04N    C04D    H04D    C02Z
              C047    H047    C04M    C02S    C039    H039    C038    H038
              C031    H031    C040    H040
PLAT790_ALERT_4_G Centre of Gravity not Within Unit-Cell: Resd.  #           5 Note
              C11 H8 O
PLAT860_ALERT_3_G Number of Least-Squares Restraints .............        1412 Note
PLAT883_ALERT_1_G Absent Datum for _atom_sites_solution_primary ..      Please Do !
PLAT909_ALERT_3_G Percentage of I>2sig(I) Data at Theta(Max) Still         58% Note
```

```
PLAT910_ALERT_3_G Missing FCF Reflection(s) Below Theta(Min)[Deg]=       3.08 Note
                0  0  1,
PLAT933_ALERT_2_G Number of HKL-OMIT Records in Embedded .res File        108 Note
               -8-10  1,  -8-10  2,  -8 -9  3,  -8 -8  4,  -7-11  1,  -7-11  2,
               -7 -9  4,  -7 -9  5,  -6-11  1,  -3-17  5,  -3-17  6,  -3-17  7,
               -2-17  1,  -2 14  5,  -1 14  4,  -1 14  6,  -1 14  7,  -1 14 11,
               -1 14 12,   0 13 14,   0 15  6,   0 15  7,   1 14  1,   1 14  5,
                1 15  4,   1 15  5,   2 14 10,   3  8 18,   3 11 14,   3 13  4,
                3 13  5,   3 14  3,   3 14  6,   3 14  7,   4 12 10,   4 12 11,
                4 13  4,   4 13  5,   4 13  6,   4 13  7,   4 13  9,   5 11 11,
                5 12  4,   5 12  5,   5 12  6,   5 12  7,   6 10 12,   6 11  1,
                6 11  2,   6 11  3,
PLAT941_ALERT_3_G Average HKL Measurement Multiplicity ..........        2.6 Low
PLAT969_ALERT_5_G The 'Henn et al.' R-Factor-gap value ..........      3.622 Note
              Predicted wR2: Based on SigI**2  6.45 or SHELX Weight 22.63
PLAT978_ALERT_2_G Number C-C Bonds with Positive Residual Density.          2 Info
```

```
   1 ALERT level A = Most likely a serious problem - resolve or explain
   1 ALERT level B = A potentially serious problem, consider carefully
  53 ALERT level C = Check. Ensure it is not caused by an omission or oversight
  46 ALERT level G = General information/check it is not something unexpected

   3 ALERT type 1 CIF construction/syntax error, inconsistent or missing data
  66 ALERT type 2 Indicator that the structure model may be wrong or deficient
  15 ALERT type 3 Indicator that the structure quality may be low
  16 ALERT type 4 Improvement, methodology, query or suggestion
   1 ALERT type 5 Informative message, check
```

It is advisable to attempt to resolve as many as possible of the alerts in all categories. Often the minor alerts point to easily fixed oversights, errors and omissions in your CIF or refinement strategy, so attention to these fine details can be worthwhile. It is up to the individual to critically assess their own results and, if necessary, seek expert advice.

Validation response form

Please find below a validation response form (VRF) that can be filled in and pasted into your CIF.

```
# start Validation Reply Form
_vrf_DIFMX02_lcz051
;
PROBLEM: The maximum difference density is > 0.1*ZMAX*0.75
RESPONSE: ...
;
_vrf_PLAT094_lcz051
;
PROBLEM: Ratio of Maximum / Minimum Residual Density ....       2.11 Report
RESPONSE: ...
;
_vrf_PLAT097_lcz051
;
PROBLEM: Large Reported Max.  (Positive) Residual Density       4.42 eA-3
RESPONSE: ...
;
```

```
_vrf_PLAT220_lcz051
;
PROBLEM: NonSolvent   Resd 3  C   Ueq(max)/Ueq(min) Range        3.9 Ratio
RESPONSE: ...
;
_vrf_PLAT234_lcz051
;
PROBLEM: Large Hirshfeld Difference F00Q     --C03S     .       0.18 Ang.
RESPONSE: ...
;
_vrf_PLAT241_lcz051
;
PROBLEM: High   'MainMol' Ueq as Compared to Neighbors of       C03B Check
RESPONSE: ...
;
_vrf_PLAT242_lcz051
;
PROBLEM: Low    'MainMol' Ueq as Compared to Neighbors of       C03X Check
RESPONSE: ...
;
_vrf_PLAT309_lcz051
;
PROBLEM: Single Bonded Oxygen (C-O > 1.3 Ang) ..........       O024 Check
RESPONSE: ...
;
_vrf_PLAT369_lcz051
;
PROBLEM: Long   C(sp2)-C(sp2) Bond  C2       - C03H     .       1.55 Ang.
RESPONSE: ...
;
_vrf_PLAT906_lcz051
;
PROBLEM: Large K Value in the Analysis of Variance ......     25.502 Check
RESPONSE: ...
;
_vrf_PLAT911_lcz051
;
PROBLEM: Missing FCF Refl Between Thmin & STh/L=    0.595        147 Report
RESPONSE: ...
;
_vrf_PLAT972_lcz051
;
PROBLEM: Check Calcd Resid. Dens.  0.85Ang From Ag08           -2.43 eA-3
RESPONSE: ...
;
_vrf_PLAT977_lcz051
;
PROBLEM: Check Negative Difference Density on H028      .      -0.37 eA-3
RESPONSE: ...
;
# end Validation Reply Form
```

PLATON version of 17/09/2025; check.def file version of 17/09/2025

---

duplicate check

No duplication found

Datablock lcz051 - ellipsoid plot

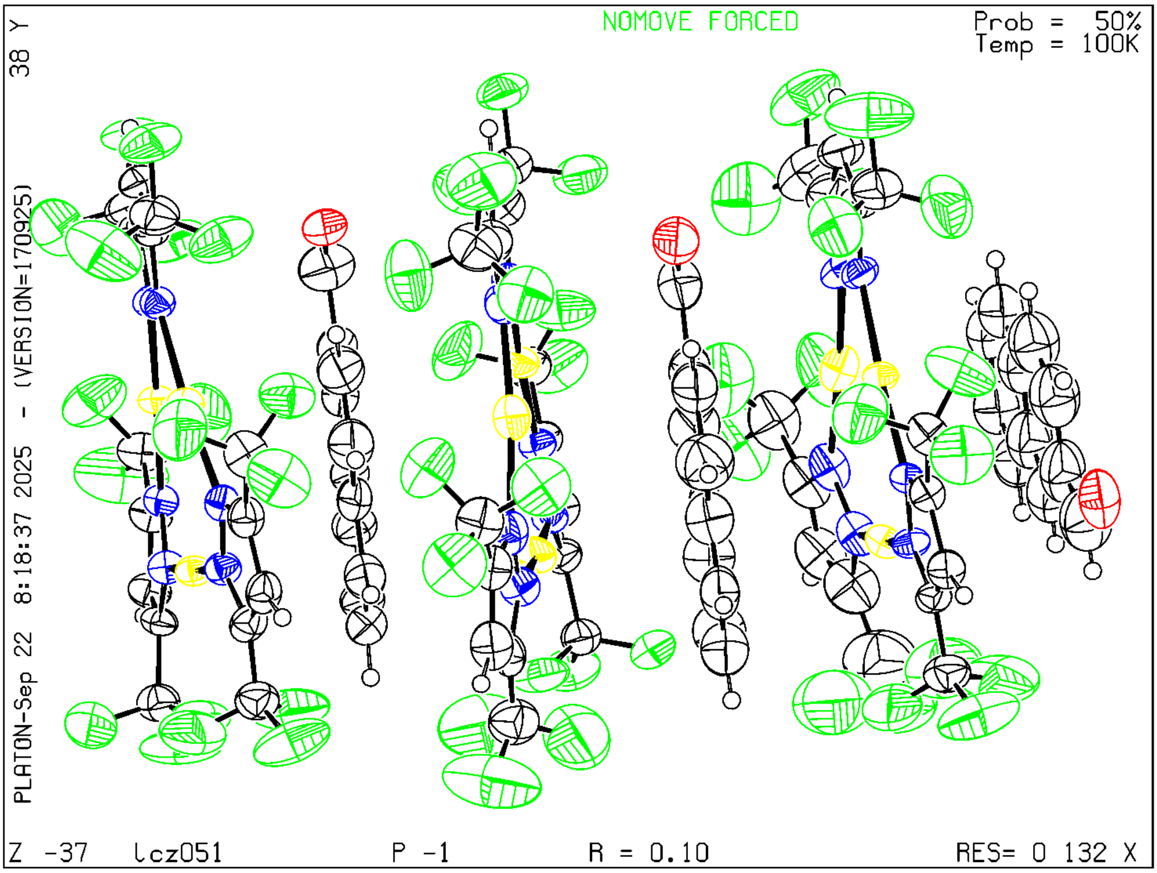

Supplement: Supplementary file 2 — Supporting File 2: anie72462‐sup‐0002‐DataFile.zip. [file ANIE-65-e1218503-s002.zip › supplementary_materials_crystallographic_data submit.zip!/cif_and_checkcif(1-114)/Ag3Pz3_51/Ag3Pz3_51_checkcif.pdf]
